# Expression profiling of heat shock protein genes in whole blood of Romosinuano cattle breed

**DOI:** 10.14202/vetworld.2023.601-606

**Published:** 2023-03-24

**Authors:** Juan Camilo Taborda-Charris, Roy Rodríguez-Hernández, María Paula Herrera-Sánchez, Heinner Fabian Uribe-García, Rafael J. Otero-Arroyo, Juan Sebastian Naranjo-Gomez, Kelly Johanna Lozano-Villegas, Iang Schroniltgen Rondón-Barragán

**Affiliations:** 1 Immunobiology and Pathogenesis Research Group, Faculty of Veterinary Medicine and Zootechnics, University of Tolima, Altos the Santa Helena, A.A 546, Ibagué 730006299, Colombia; 2 Poultry Research Group, Laboratory of Immunology and Molecular Biology, Faculty of Veterinary Medicine and Zootechnics, Universidad del Tolima, Santa Helena Highs, Postal Code 730006299, Ibagué-Tolima, Colombia; 3 Grupo de Investigación en Reproducción y Mejoramiento Genético Animal, Facultad de Ciencias Agropecuarias, Universidad de Sucre, Sincelejo 700001, Sucre, Colombia; 4 Laboratorio de Reproducción Animal, Corporación de Ciencias Biotecnológicas, Embriotecno, Montería 230029, Córdoba, Colombia

**Keywords:** climate change, external environment, heat shock proteins, heat stress, mRNA, temperature-humidity index

## Abstract

**Background and Aim:**

Heat shock proteins are highly conserved proteins that work as molecular chaperones expressed in response to thermal stress. This study aimed to determine the expression profile of genes related to the heat stress response in whole blood obtained from the Romosinuano creole breed.

**Materials and Methods:**

Real-time polymerase chain reaction was performed to analyze the transcript of *hsp90*, *hsp70*, *hsp60*, and *hsf1* in the whole blood of Romosinuano under different temperature-humidity indices (THIs).

**Results:**

The expression levels of the *hsp70* and *hsf1* genes at the high-THI level were higher (p = 0.0011 and p = 0.0003, respectively) than those at the low-THI level. In addition, no differences in the expression levels of the *hsp60* and *hsP90* genes were detected between the two THIs.

**Conclusion:**

The overexpression of *hsf1* and *hsp70* genes play an important role in protecting cells from damage induced by heat stress.

## Introduction

Climate change affects food production in tropical and subtropical regions, especially dairy and meat production [1, 2]. Adverse weather conditions limit agricultural and livestock production development and heat stress is one of the main problems in animals raised outdoors and indoors [3–7]. Heat stress in livestock is caused when environmental conditions challenge the animal’s thermoregulatory mechanisms. It is defined as the increment in body temperature that leads to a physiological response to compensate for the heat gain, avoiding a state of hyperthermia [6, 8, 9]. Heat stress has been associated with decreased milk and meat production in cattle. It has deleterious effects on reproduction and immunity due to changes in animal metabolism and physiology and is responsible for the lower quality of derived dairy and meat products [1, 8, 10, 11]. In animal husbandry, the temperature-humidity index (THI) is a parameter that considers the environmental conditions that cause heat stress, including relative humidity and environmental temperature [12]. THI allows animal classification into mild heat stress, moderate heat stress, and severe heat stress, used in performing retrospective studies, heat stress economic studies, and farm heat stress abatement strategies [8, 12].

The use of novel omics technologies is becoming increasingly common in animal production. Omics technologies are related to studying the collection of molecules, biological processes, or physiologic functions and structures as systems [13]. Transcriptomics and proteomics allow the study of animal responses at the molecular level when subjected to certain factors, including environmental conditions and diet [14]. Heat stress in cattle can be determined by identifying proteins and genes that increase their expression under heat stress, such as heat shock proteins (HSP) [5, 15, 16]. Body temperature above the threshold induces gene expression in the biosynthesis of HSP and their expression is different in each cattle breed depending on external factors [17–20]. In general, the overexpression of HSP protects against hyperthermia, circulatory shock, and cerebral ischemia during heat shock, which plays a significant role in cell protection [21]. HSP has chaperone activity that ensures the folding, unfolding, and refolding of proteins denatured by stress [22].

The survivability of cattle in the tropical environment depends on the adaptations developed throughout successive exposition to the continuous stressor, in our case, cattle raising in grazing in the tropical and subtropical regions [23]. The Romosinuano creole breed from Colombia is a breed that stands out for tolerating heat and different humidity conditions and exhibits remarkable characteristics of adaptation, such as high fertility, weight gain, and longevity, which give it an advantage over other breeds in tropical conditions [24, 25]. It has been reported that Romosinuano bovine embryos showed a better response to heat stress than other breeds, constituting an advantage for embryo development in adverse weather conditions [26]. At present, studies of gene expression profiles of genes related to heat stress in Colombian Creole cattle under tropical environmental conditions are limited. Thus, it is important to establish the gene transcripts of heat shock proteins such as *hsp60*, *hsp70*, *hsp90*, and *hsf1* in whole blood, as an additional tool for selecting better-adapted individuals in tropical conditions.

This study aimed to determine the expression profile of genes related to the heat stress response in whole blood obtained from the Romosinuano creole breed.

## Materials and Methods

### Ethical approval

All procedures involving animals were approved by the Ethics committee of the University of Tolima (Approval no. 001-2021), based on Law 84/1989 and Resolution 8430/1993 and complied with the guidelines for animal care and use in research and teaching [27].

### Study period and location

The study was conducted from July to August 2020. Fifteen healthy cows (n = 15) of Romosinuano breed (age: 48 ± 96 months) and weight (400 ± 39 kg body weight) were selected by phenotypic characteristics and productive and reproductive behavior of the parents, and located on a farm near Monteria city - Cordoba district in northern region of Colombia, (Latitude 8◦45′36′N and Longitude 75◦53′08′′W), with an average temperature of 29°C and relative humidity between 70% and 85%, in the wet season. Cows were kept under the same sanitary and nutritional management in a grazing system using *Megathyrsus maximus*, cv. Tanzania and *Dichantium aristatum*, and supplemented with corn silage, mineralized salt, and water *ad libitum*.

### Sample collection

Sixty blood samples (4 mL) were collected twice in the wet season, sampling daily between 7:00 and 9:00 h and then from 15:00 until 17:00 h. Blood samples were obtained by venipuncture of the Caudalis medium vein (tail vein) using ethylenediaminetetraacetic acid tubes (Becton Dickinson Vacutainer Systems, Franklin Lakes, NJ, USA); blood samples were divided into aliquots of 2 mL in vial tubes and frozen in liquid nitrogen, and stored at −20°C until experimental analysis.

### Weather data

Air temperature and relative humidity data were obtained from the experimental cattle yard using PCE-FWS20N weather station equipment (PCE Instruments™, Meschede, Germany). Temperature monitoring was carried out 15 days before taking the blood samples to determine the coolest and hottest hours of the day in the wet season. The THI was calculated using the formula [28] as follows:

*THI* = (1.8*Tdv* + 32) – 0.55 – 0.00551.8*Tdb* – 26 1.

THI data were used to identify categories of livestock weather safety index [29].

### RNA extraction and cDNA synthesis

Total RNA was extracted from whole blood samples using the RNA-solv® reagent kit (OMEGA, Norcross, GA, USA) according to the manufacturer’s protocol with certain modifications [28]. Before reverse transcription, all RNA samples were diluted to 200 ng/mL, and cDNA was synthesized using the GoScriptTM Reverse Transcription System kit (Promega, Madison, WI, USA) following the manufacturer’s instructions. End-point polymerase chain reaction (PCR) and agarose gel electrophoresis were performed to determine the cDNA quality and the amplicon size.

### Real-time PCR analysis

The relative expression of *hsp60*, *hsp70*, *hsp90*, and *hsf1* genes was measured by quantitative real-time PCR (qPCR) in a QuantStudio 3 Real-Time PCR System (Thermo Fisher Scientific, Waltham, MA, USA) by fast ramp program, using GoTaq^®^ qPCR Master Mix (Promega). All reactions were performed in duplicate. The relative gene expression was nor­malized using actb as a reference gene [30]. Primer sequences for *hsp60, hsp70, hsp90*, and *hsf1* amplification by qPCR assay are presented in Table-1 [30, 31]. Thermal cycling conditions were initial denaturation for 2 min at 95°C, then 40 cycles of denaturation for 3 s at 95°C, and annealing for 30 s at 60°C. Subsequently, a melting step was performed at 95°C for 1 s, 60°C for 20 s, and a continuous rise in temperature to 95°C at a rate of 0.15°C per second. The data obtained were analyzed using the 2−ΔΔCT method.

**Table-1:** Primer sequences for *hsp60, hsp70, hsp90*, and *hsf1* amplification by qPCR assay.

Gene	Primer sequence (5´-3´)	Primer length (NT)	TM (°C)	GC%	Amplicon size (bp)	Reference
*hsp60*	F GGAAAAGGTGACAAGGCTCA	20	58.02	50	214	This study
	R CAGCTCGTGTGGCATTAAGA	20	58.27	50		
*hsp70*	F AGGACTTCGACAACAGGCTG	22	56.53	39.14	141	This study
	R TGCTGGACGACAAGGTTCTC	20	60	55		
*hsp90*	F GGAGGATCACTTGGCTGTCA	20	59.38	55	177	[31]
	R GATTAGCTCCTCGCAGTT	20	59.53	55		
*hsf1*	F CCCCGACCACCCTTATTG	18	57.02	61.11	139	This study
	R GCGACGCTGAGGCACTT	17	60.42	64.71		
*actb*	F GGGATGAGGCTCAGAGCAAGAGA	23	63.65	56.52	118	[30]
	R AGCTCGTTGTAGAAGGTGTGGTGCC	25	66.91	56.00		

qPCR=Quantitative real-time polymerase chain reaction, NT=Nucleotides, TM=Temperature, GC=Guanine-cytosine

### Statistical analysis

Statistical analysis was performed using cycle threshold data from cow blood samples obtained in the morning and afternoon. The differences in the relative gene expression between the two groups were analyzed by Mann–Whitney test using GraphPad Prism software v 8.0 (La Jolla, CA, USA). Differences were considered statistically significant if p < 0.05.

## Results

### Temperature-humidity index

The coolest hours and lowest humidity percentages were between 5:00 and 7:00, with a calculated THI: 76, and the hottest and high humidity hours were between 13:00 and 14:00, showing THI: 83. Thus, blood samples were collected approximately 1 h after the lowest and highest THI, between 7:00 to 9:00 (THI: 78) and 15:00 to 17:00 (THI: 81).

### Gene expression profile of *hsp60, hsp70, hsp90*, and *hsf1*

**Figure-1:**
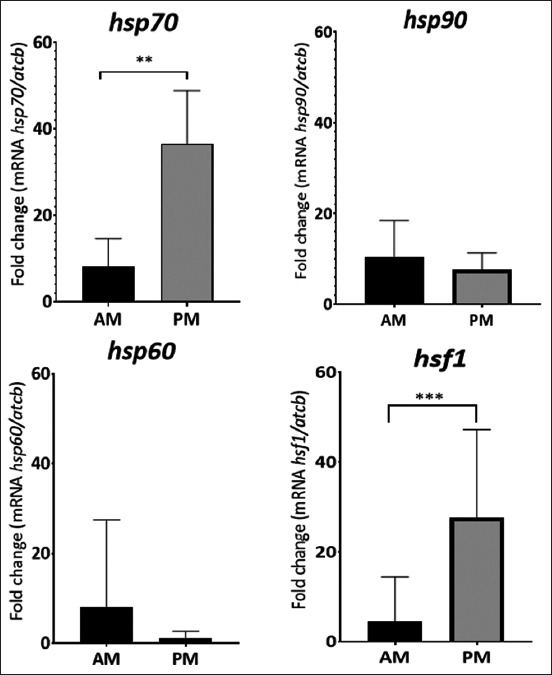
mRNA expression of *hsp60*, *hsp70*, *hsp90*, and *hsf1* in Romosinuano breed at two different hours a day. Data are presented as the means ± standard errors. Means with different superscripts differ significantly (**p < 0.01 and ***p < 0.001).

The difference between the increase in THI at the sampling times evidenced a differential expression in the *hsp70* gene (AM vs. PM p = 0.0011) and the *hsf1* gene (AM vs. PM p = 0.0003) (Figure-1). In contrast, the expression of *hsp60* and *hsp90* did not show a statistical difference between the two sampling times (p = 0.12 and p = 0.73, respectively).

## Discussion

Livestock productions are under different stressors that generate significant economic losses [32]. Heat stress is one of the most critical stressors that affect livestock production, with an estimated annual economic loss of 1.69–2.36 billion US dollars [33, 34]. Heat stress negatively affects livestock parameters, including feed intake, growth, fertility, and conception rates [35]. However, livestock can respond to heat stress through evolutionarily conserved processes that regulate differential gene expression and related pathways [36]. For example, under heat stress conditions, HSP synthesis protects cells from heat stress by repairing protein damage [37].

This study was conducted to evaluate changes in the expression of HSP genes (*hsp60, hsp70, hsp90*, and *hsf1*) in whole blood obtained from Romosinuano creole breed in two different environmental conditions during the day in the wet season, using THI according to the Livestock Weather Safety Index to categorize the heat stress associated with hot-weather conditions for livestock exposed to extreme conditions [38]. One category corresponds to the THI obtained at morning sampling, calculated THI 78 (alert), and THI obtained at afternoon sampling 83 (danger). Our results indicated that compared to the samples taken during the heat stress of the Alert category, the mRNA expression of the *hsf1* and *hsp70* genes was significantly higher than in the samples taken during the danger category (Figure-1). This way, the overexpression of *hsf1* in samples taken during the danger category can be explained because this gene is the main modulator of heat shock response [39]. *hsf1* plays an essential role in cells subjected to heat shock or other proteotoxic conditions by protecting against stressors by positively regulating HSP mRNA levels [40–42]. For example, HSF1 interaction between HSP70 and its companion HSP40 has been correlated with increased *hsp70* gene expression [43, 44].

Furthermore, the level of *hsp70* mRNA in samples taken during the category of danger can also be attributed to the conserved role that this gene plays in cellular thermotolerance between prokaryotic and eukaryotic organisms [45]. In the present study, the mRNA expression profile of *hsp70* in blood samples agrees with the previous studies, where the increment of expression of *hsp70* mRNA was reported after heat exposure at higher temperatures [46]. Further, an increase in *hsp60* expression without a significant difference was observed in the samples taken during the danger category. The induction of *hsp60* has been reported to help prevent apoptotic cell death by rapidly activating pro-caspase-3 during apoptosis [47–49]. The previous studies also reported that hsp70 and hsp60 are upregulated in cattle during heat stress [50].

On the other hand, the decrease in *hsp90* mRNA expression without a significant difference can be attributed to the rapid induction of translation of the HSP90 protein to protect cells from heat stress [51]. Finally, it is important to mention that this is the first research on HSP gene expression in the Romosinuano cattle breed. Romosinuano cattle is an adapted *Bos taurus* breed that has a thermoregulatory capacity explained through physiological processes such as the reduction of heat production and the ability to increase heat loss to the environment [52–54].

## Conclusion

Our data suggest that the THI threshold influences changes in the gene expression of some genes related to heat stress in the Romosinuano cattle in the dry tropical forest, especially in the danger THI category (afternoon) under conditions in our study and induced the expression of *hsf1* and *hsp70* genes in whole blood samples compared to the THI range in the alert category (morning), due to these proteins being in charge of cell protection during heat stress, providing information about the possible cellular adaptation in this creole cattle.

## Authors’ Contributions

JCT, RR, KJL, and ISR: Conceptualization and formal analysis. JCT, RJO, KJL, JSN, and HFU: Methodology. HFU, JCT,JSN and ISR: Software. ISR and MPH: Validation. RR, MPH, KJL, RJO and ISR: Writing – original draft preparation and writing – review and editing. KJL, MPH and ISR: Supervision. RR, JSN, RJO and ISR: Project administration. All authors have read, reviewed, and approved the final manuscript.
